# A Qualitative Study to Explore the Determinants of Risky Sexual Behaviors and Pregnancy among Female Adolescents in Sabah, Malaysia

**DOI:** 10.1155/2022/1866326

**Published:** 2022-11-28

**Authors:** Idayu Badilla Idris, Shameer Khan Bin Sulaiman, Rozita Hod, Hamed Khazaei, Nik Nairan Abdullah

**Affiliations:** ^1^Department of Community Health, Faculty of Medicine, Universiti Kebangsaan Malaysia, Kuala Lumpur, Malaysia; ^2^Malaysia-Japan International Institute of Technology, University Teknologi Malaysia, Kuala Lumpur, Malaysia; ^3^Department of Public Health Medicine, Faculty of Medicine, Universiti Teknologi MARA, Sungai Buloh, Selangor, Malaysia

## Abstract

This investigation was performed in Kota Kinabalu, Sabah state, where the highest number of pregnancies is recorded. The purpose of this study was to determine variables associated with hazardous sexual activity and adolescent pregnancy in Sabah, Malaysia. The findings indicate that familial variables, peer interactions, self-esteem, psychiatric concerns, economic considerations, and sex knowledge all play a significant role in hazardous sexual conduct and adolescent pregnancy in Sabah, Malaysia. Information obtained from this study will help the Malaysian government and other officials to design and establish proper interventions that will help alleviate the challenge of high prevalence of teenage pregnancy. It is suggested that sex education be included in the high school curriculum, along with physical and health education in Sabah, Malaysia.

## 1. Background of the Study

Teenage pregnancy is one of the social concerns in the world as well as in Malaysia. Sabah and Sarawak are showing a high rate of total teenage births, respectively [1, 2]. According to the 2011 WHO report, 15.9 million infants were born to adolescent mothers, accounting for approximately 15% of worldwide maternal mortality (Minhatet al., 2021). Despite greater efforts to educate the entire public, and adolescents, in particular, public health concerns concerning Malaysian adolescents' sexual habits continued [3, 4]. According to the state health ministry, the state of Sabah has recorded the highest number of teenage pregnancy cases, and its neighboring state Sarawak has recorded the second most teenage pregnancies in Malaysia [5]. It is startling to see that most teenagers at the age of 18 years are highly sexually active, resulting in high teenage pregnancies [5]. As Malaysia rapidly urbanizes and industrializes, the new generation not only overcomes developmental obstacles but also be prepared to deal with a plethora of environmental problems and pressures [6, 7]. Premature sexual activities among teenagers increase the risk of unprotected sex and various sex partners that may end up in sexual spread infections, such as the risk of premature rupture of membranes, acquired immunodeficiency syndrome (AIDS), postpartum infection [8, 9], and preterm labor, which may influence their study and future. Numerous risk factors for adolescent pregnancy include poverty, a lack of parental supervision, a lack of educational opportunities, and peer pressure [10]. It is therefore important to identify the contributing factors for premarital sexual behaviors among Malaysian adolescents in Sabah.

Additionally, it is considered that unintended or mismanaged adolescent pregnancy has several negative consequences, the most significant of which are on the mother and newborn's health [10, 11]. Consequently, the research study was conducted in Sabah state, where Kota Kinabalu is the capital city. The state of Sabah is divided into 23 districts, where the highest number of pregnancies in Malaysia is recorded. The objective of this study is to explore the factors contributing to risky sexual behaviors and teenage pregnancies in Sabah, Malaysia.

## 2. Methods

In-depth interviews were conducted with pregnant teenager social workers and nongovernmental organization (NGO) workers, who are directly responsible for managing teenage pregnancies, to explore other factors influencing risky sexual behavior in Sabah and get their opinions on the issue. In-depth interviews were arranged to find important factors influencing risky sexual behavior and teenage pregnancy. Respondents were teenagers, district social workers, and NGO workers in different stages in Sabah. The social workers' selection criteria were their experience in handling teenage pregnancies. They asked about the factors mentioned in this study and if they can contribute more according to their experiences with risky sexual behavior and teenage pregnancy. Interviewees were 4 teenagers, who are under the government clinic follow-up, 2 district social officers (Semporna and Kota Kinabalu) and 2 nongovernmental organization (NGO) workers. Invitations were initially distributed to prospective participants via formal and informal (friends and community members) networks. Participants were invited to schedule the interview time that was convenient for them. Open-ended questions and follow-up interviews were used for this study to determine how respondents perceive the factors related to this study and what is especially significant to them, what might be significant to others, and how it came to be what it is(Krathwohl 1998; Symon and Cassell 2012; [12]. In choosing the sample of participants, the researchers used a purposive sampling method. The study used thematic analysis to identify, analyze, and interpret qualitative data [13, 14].

### 2.1. Interviewee Profiles

Participants included executives from eight respondents. In-depth interviews were conducted with 4 adolescent girls (two of them had sexual activity experience) residing in Sabah, 2 government social workers and 2 NGO employees who were experienced in dealing with teenage pregnancy issues in Sabah. To maintain confidentiality, all participants were assigned pseudonyms. Interviews were digitally recorded and verbatim transcribed.

### 2.2. Family Factors

According to the results, teens that have difficulties in their new communities due to a lack of extended family, social, and cultural networks are more likely to engage in risky sexual behavior. Family and friends are viewed as a source of support, which is critical for youngsters' reintegration into education, job, and social life. Some of the young mothers according to respondents in this study traveled alone, while others traveled with extended family and friends. Teens that had a positive relationship with their parents or guardians were more likely to receive support, even consultation.

*My mother work[s] very hard [to ensure] that…… I can go to……..to college, so if I am pregnant will ……. disappoint my mother. The fact that so many girls are dropping out because they have children. I…….. do not want to…….. happen to me. My mother is familiar with my friends, and ……. we are comfortable with them... (R2).*

Teenagers who lived with a biological parent or first-degree relative received significantly more assistance and support from their partners than those who did not live with their parents or relatives. Teens living with guardians stated that they would have received better assistance if their biological mothers were present.

*“Is not a good thing…. I know…, but she's not good thing [stepmom] She ….. she ignores me in the house. Even if she see…. see me on the street, she [does] not say hello. This is not good. Thus, I like to have my mom (R1).”*

Mothers played a lead role in supporting teens and preventing them from early sexual behavior, even if they were sad or dissatisfied with their daughter's needs. Participants indicated that mothers can support teens because teen mothers usually feel a sense of duty toward them. Their own mothers' actions of kindness toward young mothers and their infants were highly regarded and recognized. Several interviews revealed the participants' mothers' and families' support. Young mothers' intentions and capacity to re-engage with education and work were significantly influenced by the level of help they got. Teenagers who received greater support from their families, particularly their moms, were more likely to return to school or express an interest in returning.

*“My mother once asked me, “Are you having sex?' and I replied, ‘Yes.' My mother was concerned with my studies and other activities, and…….. I…… then I speak [spoke] with my mother and we had a short fight... then she says I have to focus on school....... As soon as school began….. I returned to school, simply because I like it. I desired to achieve something beneficial for… myself when I …..I am adult. (R2).”*

Moreover, other siblings were helpful and supportive. Another teen who had risky sexual behavior obtained assistance from their sisters in asking for information, allowing them to be more careful at school or society.

*“I [had] learned from her [sister] to… [be] careful not to have children…. while still in school or out of home (R3).”*

Some teenagers who had fathers in Sabah were unconcerned about obtaining their fathers' assistance, for those whose fathers were missing from their lives in some capacity or another, which frequently resulted in loss of emotions. One out of two teenagers was from a single-parent household, and another had a father who had frequently married other wives.

*“I tried to be near him,……but he is busy or, he is always thinking [about] elsewhere. I'm sad (R4).”*

Lack of family care and teenage ignorance are also identified as contributing components. Even though teenagers are aware of preventive measures, they do not consider repercussions, demonstrating their ignorance. Schoolgirls are growing up in a society with a high proportion of parental unemployment, which is a challenge for them.

*“As I said, the factors are numerous,…….that [is] including education in the home, which occurs when parents do not recognize the importance of education, unemployment, which results in poverty,……. and poverty, which occurs when learners,…… in the majority of cases, are forced into relationships that result in pregnancy due to the inability of their parents to meet their material requirements. (R5)”*

*“In fact,…….. according to my own beliefs, I………I should make a contribution to the environment itself. Well, the circumstance itself in certain homes, when parents do not care for their children and instead go out to ….. for the entire day, does contribute to this. (R4)”.*

### 2.3. Peer Relationships

Respondents discovered that pregnant teenagers have usually negative interactions with their peers.

The explanation for this is that learners who are not pregnant view themselves as fortunate not to fall for the trap, undermining those who are pregnant. Pregnant learners, respondents report, frequently suffer from inferiority complexes, lack of confidence in the sense that others are laughing or gossiping about them, and low self-esteem. Others exhibit a reserved demeanor or are unwilling to associate with their peers, as seen by instructors.

Subthemes pertaining to the poor relationships between pregnant teenagers and their classmates are also highlighted based on the data gathered during interviews. For instance, violent behavior and a lack of confidence are all common characteristics of pregnant teenagers. The following spoken quotations substantiate the above findings:

*“Some experience, hm..., pregnant bad mood, while others exhibit unpredictable behavior at school (R3).”*

*“They argue and fight,….. perhaps because they are stressed in some way (R7).”*

*“Eh..., from what I've noticed thus far, some people feel lonely as they begin to withdraw themselves from others, from their peers (R4).”*

Peer pressure is a term used to describe the pressure that others exert on one another. Teenage pregnancy occurs as a result of a variety of interrelated factors. In some studies, peer pressure is at the top of the list of factors that contribute to pregnancy (Naong, 2011).

The participants in this study also argued that peer pressure plays a significant role in risky sexual behavior because teens are eager to satisfy their peers or even engage in sexual activities in order to acquire admission to certain social groups.

*“They become pregnant because they…… they want to be able to have sex in the same way that their friends have. As you can see, a group of teens may all have boyfriends or even become pregnant at the same time (R6).*

*“ They don't mind that they are expecting a child. If you are not with someone or engaged in sexual activity, you cannot be their friend. Friends will have an impact on your behavior (R6).”*

In light of these facts, it is impossible to ignore the importance of peer interactions in the decision-making process about sexual health. Friendships fall within the interpersonal level of society, which can result in teenage pregnancies. This level provides an individual with a sense of social identity, support, and role description. Some teenagers derive their social identity and acceptability from their classmates, who play a vital role in the formation of their social identity.

*“Usually, when I…. I am new in school, eh...., I occasionally feel alone (R2).”*

Peer pressure can be avoided by seeking advice from family and friends. Friends are valuable, but they may also be deceiving.

*“I like to ….. to be …. with my friends,….. because they are a part of me,….. they are also not... my friends [are] having sex,…….they ….. tell me what they are doing and may even ask me to their homes (R2).”*

Instinctively, peer pressure is a bargaining point because it can be either negative or positive. Teenagers who do not want to be labeled “queer” need social approval within peer groups to survive. As a result, they are pressured to join some of these organizations, and as a result of a lack of sufficient sexual health education, they become pregnant and prone to risky behaviors.

Although some teenagers are more susceptible to peer pressure than others, others have developed strategies for dealing with the negative effects of their peers. The importance of family in providing support and offering advice cannot be overstated.

*“You cannot make the decision to have sex solely based on your buddies having sex. It's crucial to know your background. What has your mother, or perhaps your grandmother, taught you through the years? Any problems should be brought to the attention of your mother, who is far more experienced and will always know what to do (R3).”*

Respondents argue that teenage pregnancy can be the effect of a lack of support in seeking empathy. They have asserted that when teenagers feel alone and rejected, they are more likely to engage in risky relationships at the early stage.

*“There are so many factors why teenagers do that. I believe teenagers get engaged because of lack of love, or feeling alone relation to community, and they don't get enough friendship from their relatives or friends. They don't get enough love and they get it from other people like the opposite sex (R5).”*

Some participants mentioned the usage of alcoholic substances or sexual abuse as the potential cause of risky sexual behavior.

*“Some of them mingling around with their peers, boyfriends, playing…….., involved in substance abuse, and some were being forced to have…… you know……. sex. These factors lead to early pregnancy (R7).”*

*“She, drank Beer. Her boyfriend then handed her medication to terminate the pregnancy. (R5)”*

*“Every one has…boyfried I guess they…… think it would make their boyfriend stay. If you are with someone or not have sex…., you cannot be their friend (R2).”*

### 2.4. Self-Esteem

Interviews also stressed the importance of self-assurance, self-control, and confidence associated with risky sexual behaviors. Respondents found that adolescents who have a negative reaction to stigma are more associated with pregnancy during secondary school. Respondents have identified behavioral trends associated with risky behaviors. They asserted that these behavioral tendencies are the result of pregnant students' refusal to embrace their motherhood state, which is exacerbated by their diverse personality features. The following spoken quotations substantiate the above findings.

*“Mmmm..., the issue…… is that if the youngster is vulnerable when lacks confidence, she may frequently believe that her peers are mocking her, so seek for having risky relationship (R3).”*

*“Well, actually, the majority of them are extremely sad for some events, as the majority of their pregnancies are unplanned, and as a result, they are extremely remorseful and will isolate themselves from other learners (R7).”*

*“Perhaps learners, particularly those of today, appear to perceive no difficulty, or perhaps it's something to have a relationship in order to qualify for the award (R6).”*

*“Some,…… develop a defense mechanism,…… others become weak, and others may find ways to confront hardships (R8).”*

Respondent information indicates that an adolescent might exhibit apologetic behavior toward their actions. They stated that some students would suddenly become quiet at school despite the fact that they are known to be talkative; this was especially true after they discovered they were pregnant. The following verbal quotes substantiate the abovementioned difficulties raised by respondents.

*“To be sure, few students will suddenly become quiet in class. They later found [to be] pregnant (R5).*

Respondents discussed depression, stating that learners who have recently given birth have a general sense of unworthiness to be considered school learners in the same way they were previously. As a result, they become shy or reserved, lonely and feel excluded, are unable to concentrate in class, are absent-minded or passive, and experience feelings of insecurity.

*“Those students who are not active,…… or involved in class, they are vulnerable. You can see that this individual requires assistance (R7).”*

*“The vulnerable teens might deny having trouble or fear of something. You'll discover that even if she is quiet at school,……. she will also do not talk too much at home (R6).”*

*“I believe that this may cause some teenagers to deny their pregnancy. There is fear or apprehension about the child's responsibilities... They adamantly dispute their demise (R3).”*

A lack of parental support and a lack of confidence might cause them to seek a boyfriend or partner who should be providing some form of support to the teenager.

*“Many times,…… I feel alone……. I feel no one like[s] me. I feel….. I am not good. I want someone to like me (R2).”*

### 2.5. Psychological Issues

Negative emotions and psychological difficulties have been demonstrated to be issues produced by anxiety and stress disorders, which are associated with learning difficulties, low self-esteem, and a sense of vulnerability to dangerous actions.

Distress might be a powerful predictor of teenage pregnancy and, during and after pregnancy, can lead to depression, social marginalization, low self-esteem, and poor academic performance in adolescents.

*“Most of the pregnant teens appear to be stressed, despondent, impatient, violent, and uncared for, and they lack the sense of adolescence (R4).”*

*“The student's anxiety and subsequently passive behavior in class demonstrate that this learner requires urgent assistance (R5).”*

*“They can be visible even in class. When,………. a good learner is …..is no longer performing well in class,……she is withdrawing from all activities,……and is attempting to withdraw from everything,….. that is the bad sign (R6).”*

*“The majority of them uh..., they exhibit signs of sadness and become hostile. As you can see, these students are depressed (R3).”*

*I was very sad…. I found a boyfriend…I [was] not thinking about what happens [next]. (R1).*

Young girls explained how being involved in risky sexual activities might affect their educational or professional opportunities. However, for the majority of them, this disturbance is not prior or they do not have any hope for the future. Several of them expressed their dissatisfaction with their life.

However, respondents indicated a dilemma between having early and risky sexual behavior and pursuing school or a career in order to avoid becoming reliant on handouts. They emphasized the critical nature of thinking about the future while they were small.

*“Perhaps learners, particularly those of today, appear to perceive no good future, or perhaps it's something to have a relationship in order to qualify for the award (R6).”*

*“I just want to see …..try it…, but I wish I….[could have] waited... I've just tried it. To …..I …..was not good about doing it. My boyfriend…..asked me to do so. I did not think of what is next, I don't think for [my]…. future (R1).”*

*“I have nothing [no plan] for….. for,….. my future (R2).”*

### 2.6. Economic Factors

The findings from both NGO and government employees' responses revealed that poverty is a contributing element. Because the teenager's parents are not receiving much, teens are looking for a connection with men who are employed and have financial resources.

*“Poverty is one of the main factors. They are vulnerable if… if they still rely on the help of….. the others, such as those…… those who tell them they love them, bring them food, and encourage them to sleep even when they don't feel like it (R7).”*

*“Because…. there are so….. so few specialists in this field, after students graduate from high school, they believe….. they have done their best. It is my belief that more awareness campaigns on adolescent learner pregnancy, as well as regarding their future, will encourage them to create objectives for themselves. Nothing now interests them because there is no money to pursue higher education (R6)”*

Risky sexual behavior or adolescent pregnancy is therefore associated with a cycle of poverty, in which very young moms remain poor and their children go on to suffer teen pregnancy as well as poverty and poor academic achievements as adolescents themselves.

*“You ….. see…. there are very few educated individuals in this area; as you look……, you will notice that they take children to school, both boys and girls drop out for the simple reason that they do not have the financial means to take them…… farther or even to support them with school needs (R8).”*

They also become a burden on their families and society because of their poor academic performance at school, which places a limit on their educational and economic stability. It is believed that when schoolgirls miss a few days of school due to poverty, the short-term disruption of their education will result in long-term underachievement, which will lower the school's pass rate over time.

*“In this area……we can't get the …. Books or school things……, such as school uniforms. My parents……. Have no money (R1).”*

*“It is possible to allow these trainees to go after working individuals or those who have money, such as taxi drivers, making it easier for the guys to sleep with them and have children (R3).”*

### 2.7. Sex Knowledge

Respondents argue that teenage pregnancy can be reduced or prevented by education. They have asserted that teenage pregnancy can be prevented, citing the significance of educating youngsters about the value of education and their future. Numerous respondents suggested strategies for reducing teenage pregnancy, including integrating sex and health education into secondary and primary schools, conducting risk of early pregnancy and HIV awareness campaigns, and networking with or inviting other community stakeholders, such as social workers, health workers, and school psychologists, to speak to learners about pregnancy and its effects. The following verbal quotes substantiate the issues raised by respondents.

*“Sex education is important,…… sure it can be prevented or decreased by teaching sex education as early as possible, perhaps as early as in the first Grade (R3).”*

*“I think yes….. so, because we know about the problems…., and then we ask (R2).”*

*I believe so, because we educate kids on a regular basis through education, and then through the media, which also plays an important part (R4).*

Respondents believe that those students who have not yet begun sexual relationships, such as those in the phase of secondary school, should be guided by various professionals who would be invited to school to speak about teenage pregnancy to delay engaging in sexual intercourse or even abstain from sex until they get married, rather than waiting until they get married.

*“I think if there are some … programs in the….. churches or mosques, they could get help or be guided to according to the ceremonies or ….. or through …… the counselling and guidance (R6).”*

*“Through the……. participation of social workers and health advisors from all the clinics we're working to… to debate the recommendations that will be used, as well as representatives …..from the hospitals and psychologists, we're able to get a more effective outcome (R6).*

It is also highly encouraged by instructors that students use contraceptives. Students who have reached the stage of engaging in sexual relationships, which refers to those in the phase of secondary school, should take preventative education about using medications and condoms.

*“Yes,….. I believe …..we are capable of preventing teenage pregnancy. ……Learners must be taught in school that prevention is always preferable to cure when it comes to health problems (R8).”*

*“I believe the…… best course of action is to..., to tell those who are learning to refrain, but for those who are already doing so, they can use condoms or perhaps some tablets to keep it from happening again (R6).”*

Teens are likely to not use condoms or even birth control pills, which are efficient in preventing pregnancy. Some show shyness to go to the drugstores and buy them, and some when not taken consistently and properly.

Their lack of preparedness and lack of awareness about risky sexual behaviors such as unwanted pregnancies prompted the teenager to behave rashly and frequently incorrectly with the aim of keeping their partners happy. They might face psychological strain as a result of their familial difficulties. Additionally, when boys do not care about contraceptives, they seem to follow.

*“Because my guy….. don't likes …….condoms, we did not use them (R1).”*

## 3. Discussion

### 3.1. Family Factors

Consistent with the outcome of this study, Sprecher et al. [15] also found family background variables to be associated with the perceived acceptability of the timing of early sexual activity. Salasiah et al. [16] organized a questionnaire-based survey among fifty pregnant teenagers at two women's sanctuary houses and found that almost fifty percent of the respondents agreed that lack of proper communication with their parents hindered them from talking about their troubles. Moreover, most teenagers' parents were busy at work, which resulted in a lack of awareness and supervision of their children. Another focus group research by Jamaludin et al. [17] found the same family communication issue among pregnant teenagers. Excessive freedom and lack of devotion and love from parents led those adolescents to engage in too early sexual experiences and become pregnant. In a study by Akbari Kamrani and Yahya [18], the authors determined the cause of data concerning sexual and reproductive health among girl students in the Klang Valley, Malaysia. They discovered that parents were the key or main source of sex-related knowledge, and nearly half of the teenagers had never debated sex-related matters with their parents, while only 6.3 percent of them talked about them frequently with their mothers.

### 3.2. Peer Relationships

Teenage girls are stated to relate their experiences of intercourse to their influence from peers and society [19]. Similarly, in another study, teenage females were reported to compare their intercourse experiences or sexual behavior with their peers, as everyone else was doing it [20]. Adolescents face a variety of issues as a result of their vulnerability and distinctive features, including juvenile delinquency, sexual exploitation, violence, and abuse [21].

In some cases, there was pressure on them for sexual consent to obtain peer endorsement and not want to leave them behind [22]. The influence of group, society and peers on teenagers may increase the risk of unhealthy sexual behavior [23, 24].

In China, for instance, teenage girls whose peers were living with boyfriends or working at entertainment places were two times more expected to have a higher risk of unhealthy and unsafe sexual behavior [25]. Having a minimum of two friends who use contraception has been stated to decrease the odds of pregnancy among teenagers [26]. It seems that young males are experiencing more peer pressure than females in terms of engagement in sexual behavior [23].

Social support is described as emotive and influential support from family, peers, and society, which has an essential influence on an individual's rational behavior [27]. In a study conducted in Mexico, perceived social support from family members, school teachers, friends, neighbors, and other adults was found to be a hindrance to sexual activity among middle school students [28]. However, the outcomes of sexual activity cannot be clarified by social support if they are associated with forced sexual activity specifically at an earlier age. Norhasmah [29] also argued that social support and regularity of peer contact are linked with physical relationships and sexual activities among teenagers in Malaysia.

### 3.3. Self-Esteem

Self-esteem is an individual's subjective evaluation of their worth. Self-esteem encompasses beliefs about oneself [30, 31]. Kaplan et al. found that pregnant teenagers had lower original self-esteem than nonpregnant teens. Nevertheless, there are conflicting results when assessing pregnant teenagers' self-esteem. It appeared that pregnant teenagers' self-esteem depends on numerous factors, including school enrollment, perceptions of the pregnancy event, and accomplishments [32].

Moreover, attitude toward school is positively related to educational levels, teenage prospects, mother's and father's educational levels, and age [33]. Therefore, teenagers with higher self-esteem were more likely to have a positive attitude toward their schools and have more self-efficacy and have less traditional viewpoints of women's roles in the family. Pregnant teenagers with conservational viewpoints of their roles in the family were more likely to have lower school attitudes, hope, and age at first pregnancy [34]. Furthermore, these teenagers have been raised in deprivation and disapprove of the pursuit of a career [33].

Teenage mothers usually demonstrate more identity diffusion, less autonomy, coping difficulties, and a low level of self-esteem [30]. Moreover, teenagers with higher self-esteem, who have not been raised in poverty, have a more internal locus of control, and have less traditional views of women's roles in the family. An internal locus of control is positively related to expectations and educational levels, the mother's and father's educational levels, and age at first pregnancy [33] .

Self-recognized efficacy is the extent that the individual's beliefs about their ability to control events that affect his or her life. This construct is a fundamental factor in human belief, motivation, performance behavior, and emotional well-being [35, 36]. A study by Coyle et al. [37] supports the hypothesis that teenagers with high self-efficacy think that they can and should be responsible for their sexual activity and act appropriately to achieve contraceptive safety. Teenagers with low self-efficacy may fail to use effective protections and experience conflicts regarding their strong sexual experiences and feelings.

### 3.4. Psychological Issues

Depression is described as a frequent and serious mental illness that affects a person's feelings, thinking, and actions negatively. Depression is also called a depressive disorder and generally happens at any time. Women are more vulnerable to depression before and after childbirth. Depression disorder is expected to happen in roughly 10 to 20 percent of new mothers [38].

Earlier studies argued that depression is one of the most significant predictors of premature pregnancy and can be moderated by other sociodemographic traits [39]. However, few studies found that postpartum depression is also related to marital status as four out of nine (44.4 per cent) single women exhibited depression [40]. Furthermore, Räisänen et al. [41] argue that there is a double risk for mental problems with unintended pregnancies among unmarried women because of low support from family and society.

Therefore, it is important to help teens develop their consciousness to solve personal, interpersonal, and psychological issues. This will aid them in tolerating, mastering, and reducing the stress which might lead to risky sexual behavior [29, 42, 43].

### 3.5. Economic Factors

Moni et al. [44] discovered that teenagers with low socioeconomic levels and a smaller number of school years were at four times greater risk of pregnancy. Compared with those teenagers living with their parents, those who are alone and unsupported throughout high school will have more risk of early sexual practices [45]. A parent's socioeconomic status is also related to adolescent sex behavior [20] and sexual well-being [46]. Those with jobless parents or living in poverty showed a higher risk of early sexual behavior and that can be one of the ways to make money [45]. Parental deprivation may be a pushing factor to exposing teenage females to early sexual behavior when engaging in society [19]. Moreover, a parent's lower educational level also increases the probability of early pregnancy, and the mean years of education of parents were found to differ between women who experienced teen pregnancy and those women who did not [47]. Teenagers who live in households with a low level of education and high residential turnover are at a greater risk of teenage pregnancy [48]. Acharya et al. [48] explained that educational attainment, socioeconomic status, cultural factor, and family formation were all identified as factors affecting risky sexual behavior and teenage pregnancy in South Asia.

### 3.6. Sex Education

Sex education is found to be among the factors influencing healthy behavior in Sabah. Education motivates individuals to accept health-promoting programs and helps people make better decisions. It may bring some shift in beliefs and attitudes or influence values and facilitate the acquisition of skills and affect changes in lifestyle and behavior [49]. Efficient education will also produce a positive change in understanding the consequences of unhealthy behavior [50]. Mushwana et al. [51] studied factors influencing the adolescent pregnancy rate in the Greater Giyani Municipality, Limpopo Province, South Africa. They discovered that insufficient sexual understanding is a significant factor contributing to the high pregnancy rate among teenagers.

Sex education is one of the most crucial sources of teenagers' health knowledge that requires inspiration to implement healthy sexual behavior and help them make proper decisions. Efficient sex education will enhance their knowledge and bring some shift in beliefs and attitudes toward sexual behavior [52]. Proper reproductive and sexual health education can be integrated into the school curriculum, and its elements are being taught throughout current courses, such as health and physical education, ethical and Islamic education, science, and biology. Religious and cultural factors in Malaysia require no legal obligation for sexual knowledge education in the school system. According to cultural and religious concerns, sexuality in this country is deemed a sensitive subject [53]. As a result of this sensitivity, teenagers are not receiving adequate education, information, or guidance on reproductive and sexual health. Inadequate understanding of their sexuality and bodies makes them vulnerable when involved with unexpected and undesirable sexually transmitted diseases and pregnancy, as well as unsafe abortions [54]. A lower degree of sex knowledge, especially about the function of contraception and their reproductive organs, makes teenagers exposed to a higher risk of unintentional pregnancy [55]. According to a study by Nordin et al. [56], school teachers, media, parents, and related organizations require to deliver fundamental information to teenagers, particularly girls about their risky sexual behavior and relationship with their partners, as well as the outcomes of an unnecessary pregnancy.

Information about reproductive health among teenagers has an important impact on the welfare of both teenagers and society. A lack of or insufficient sex knowledge may cause a serious problem by exposing them to various risks to their well-being [57].

## 4. Conclusion and Recommendation

Due to their susceptibility and distinguishing characteristics, adolescents encounter a multitude of difficulties, including juvenile delinquency, sexual exploitation, violence, and abuse. Teenagers undergo significant and painful changes in their biological, physical, emotional, social, and economic life as they transition from childhood to maturity. At this vital period of development, adolescents form and acquire habits, behavioral patterns, and lifestyles that will last a lifetime. These habits will affect the well-being of future generations. The advantages of any society that addresses the issue of promoting teens' growth much outweigh costs associated with neglecting their demands. This research was conducted in Sabah state, where Kota Kinabalu is the capital city. The objective of this study was to explore the factors contributing to risky sexual behavior and teenage pregnancy in Sabah, Malaysia. The results show that family factors, peer relationships, self-esteem, psychological issues, economic factors, and sex knowledge are the most important factors contributing to risky sexual behavior and teenage pregnancy in Sabah, Malaysia.

Sex education, along with physical and health education, may be introduced into the high school curriculum. Malaysia's Ministry of Education may encounter several conflicts in administering this program and achieving its desired goals. In practice, sex education should also be allocated to schoolteachers who may lack adequate training in this area.

Implementing a comprehensive national sex education program would be a time-consuming task for the Malaysian government. Malaysia's diversified community may easily disapprove of school-based sex education.

### 4.1. Ethical Considerations

This study has been approved by the Ministry of Health Medical Research Ethics Committee (MREC), Ministry of Health Malaysia (Approval Document Number: NMRR-20-3121-56066). The researcher also asked for verbal consent from teenagers or parents or guardians of respondents of this study. The written informed consent considers any upcoming uses of data and how data were collected, protected, and utilized over a long period. Privacy was preserved by eliminating direct identifiers (names) by replacing them with assumed names to report, share, and publish the outcome of the study.

### 4.2. Implications of the Study

According to the state health ministry, the state of Sabah has recorded the highest number of teenage pregnancy cases, and its neighboring state Sarawak has recorded the second most teenage pregnancies in Malaysia. Underprivileged situations may cause many teenagers to have unhealthy sex activities or become pregnant. Although the deprived environments of most teen mothers account for many of the burdens that these adolescent women hold, having a baby or sexual diseases during their teenage years constrains economic and educational opportunities. Therefore, the current study intends to investigate conceivable factors influencing this dilemma, and subsequently, it proposes procedures to prevent the consequences of risky sexual behavior and teenage pregnancy in Sabah. Moreover, it is believed that officials and the government will use the outcomes of this research to establish proper resolutions that will help alleviate the dilemma of teenage pregnancy in Sabah. By finding the root causes of the problem, this study will help manage the situation that causes teenage girls to become pregnant, hence reducing consequences associated with it. Teenage pregnancy and sexually transmitted diseases are documented as serious concerns in society regardless of initiatives carried out so far. Information obtained from this study will help the Malaysian government and other officials design and establish proper interventions that will help alleviate the challenge of high prevalence of teenage pregnancy in Sabah, Malaysia.

### 4.3. Limitations of the Study

A limitation of this study was that it collected data from only residing adolescents in Sabah, Malaysia, eliminating data from adolescents and experts who did not reside in Sabah. Moreover, the objective of qualitative research is not to generalize but rather to gain a comprehensive understanding of the phenomenon, considering a small sample. Therefore, the study's conclusions might not be generalized to the whole of Malaysian society [58, 59].

In addition, adolescent pregnancy and sexual behaviors are highly sensitive topics; consequently, talks could have resulted in participants withholding crucial information from the researcher or even withdrawing from interviews. The participants may have also provided the information that they believed the researcher would want to hear. These constraints tried to be overcome by informing participants that their candor would be crucial for recommending targeted treatments to minimize adolescent pregnancy by assuring their anonymity.

## Data Availability

The participants of this study did not give written consent for their data to be shared publicly, so due to the sensitive nature of the research, supporting data are not available.

## References

[1] Edgar O. (2015). *Teen Sexuality in Sarawak and Sabah Highest in Malaysia*.

[2] Awang H., Low W. Y., Tong W. T. (2018). Differentials in sexual and reproductive health knowledge among east Malaysian adolescents. *Journal of Biosocial Science*.

[3] Zain N. M., Low W. Y., Othman S. (2015). Factors associated with pregnancy among unmarried women in Malaysia. *Southeast Asian Journal of Tropical Medicine & Public Health*.

[4] Abdullah S., Ghani S. A., Sipon S., Akil S. M. S., Faudzi N. M. (2014). Relationship between parent and peer attachment with coping strategy among teenagers pregnancy. *Procedia - Social and Behavioral Sciences*.

[5] Panting A. J., Abdullah H., Roslan S., Ismail I. A. (2019). Potential social risk factors for teenage pregnancy in Sarawak. *Pertanika Journal of Social Sciences & Humanities*.

[6] Khazaei H., Tareq M. A. (2021). Moderating effects of personal innovativeness and driving experience on factors influencing adoption of BEVs in Malaysia: an integrated SEM–BSEM approach. *Heliyon*.

[7] Khazaei H. (2019a). *Influence of Career Development and Training Budget on Employee Retention in Manufacturing Sector in Penang Malaysia*.

[8] Lottes I. L. (2002). Sexual health policies in other industrialized countries: are there lessons for the United States?. *The Journal of Sex Research*.

[9] Miner M. H., Coleman E. (2013). Compulsive sexual behavior and its relationship to risky sexual behavior. *Sexual Addiction & Compulsivity*.

[10] Sulaiman S. K., Hod R., Idris I. B. (2021). The effects and consequences of uncontrolled teenage pregnancy in Sabah, Malaysia. *International Journal Of Progressive Research In Science And Engineering*.

[11] Males M. (1992). Adult liaison in the “epidemic” of “teenage” birth, pregnancy, and venereal disease. *The Journal of Sex Research*.

[12] Evans C., Lewis J. (2018). *Analysing Semi-structured Interviews Using Thematic Analysis: Exploring Voluntary Civic Participation Among Adults*.

[13] Sundler A. J., Lindberg E., Nilsson C., Palmér L. (2019). Qualitative thematic analysis based on descriptive phenomenology. *Nursing open*.

[14] Braun V., Clarke V. (2021). *Thematic Analysis. Analysing Qualitative Data in Psychology*.

[15] Sprecher S., O’Sullivan L. F., Drouin M., Verette-Lindenbaum J., Willetts M. C. (2022). Perhaps it was too soon: college students’ reflections on the timing of their sexual debut. *The Journal of Sex Research*.

[16] Salasiah H. H., Al-Adib S. M., Rosmawati M. R., Fariza M. S. (2012). Factors relating to premarital pregnancy amongst muslim adolescents in Malaysia. *Research Journal of Medical Sciences*.

[17] Jamaludin Z., Bakar Z. A., Abdullah W. A. R. K. W. (2013). Adolescent pregnancy: factors and solution in Islamic perspective. *Advances in Natural and Applied Sciences*.

[18] Akbari Kamrani M., Yahya S. S. (2016). Bringing X, Y, Z generations together to facilitate school-based sexual and reproductive health education. *Global Journal of Health Science*.

[19] Ankomah A., Mamman-Daura F., Omoregie G., Anyanti J. (2011). Reasons for delaying or engaging in early sexual initiation among adolescents in Nigeria. *Adolescent Health, Medicine and Therapeutics*.

[20] Wang’eri T., Otanga H. F. (2013). Family, peer and protective factors related to sex behavior among urban adolescents in secondary schools in Mombasa County, Coast Province, Kenya. *International Journal of Education and research*.

[21] Hazariah A. H. S., Fallon D., Callery P. (2021). An overview of adolescents sexual and reproductive health services provision in Malaysia. *Comprehensive Child and Adolescent Nursing*.

[22] Skinner S. R., Smith J., Fenwick J., Fyfe S., Hendriks J. (2008). Perceptions and experiences of first sexual intercourse in Australian adolescent females. *Journal of Adolescent Health*.

[23] Bhatta D. N., Koirala A., Jha N. (2013). Adolescent students attitude towards premarital sex and unwanted pregnancy. *Health Renaissance*.

[24] Sieving R. E., Eisenberg M. E., Pettingell S., Skay C. (2006). Friends’ influence on adolescents’ first sexual intercourse. *Perspectives on Sexual and Reproductive Health*.

[25] Yan H., Li L., Bi Y., Xu X., Li S., Maddock J. E. (2010). Family and peer influences on sexual behavior among female college students in Wuhan, China. *Women & Health*.

[26] Majumdar D. (2006). Social support and risky sexual behavior among adolescents: the protective role of parents and best friends. *Journal of Applied Sociology*.

[27] Pushkarev G. S., Zimet G. D., Kuznetsov V. A., Yaroslavskaya E. I. (2018). The multidimensional scale of perceived social support (MSPSS): reliability and validity of Russian version. *Clinical Gerontologist*.

[28] Reininger B. M., Pérez A., Aguirre Flores M. I., Chen Z., Rahbar M. H. (2012). Perceptions of social support, empowerment and youth risk behaviors. *Journal of Primary Prevention*.

[29] Norhasmah (2016). Outcomes of pregnancy among unmarried mothers in Malaysia. *Doctoral Dissertation*.

[30] Goossens G., Kadji C., Delvenne V. (2015). Teenage pregnancy: a psychopathological risk for mothers and babies. *Psychiatria Danubina*.

[31] Gray-Little B., Williams V. S. L., Hancock T. D. (1997). An item response theory analysis of the Rosenberg Self-Esteem Scale. *Personality and Social Psychology Bulletin*.

[32] Ciarrochi J., Heaven P. C. L., Fiona D. (2012). *The Impact of hope, Self-Esteem, and Attributional Style on Adolescents’ School Grades and Emotional Well-Being: A Longitudinal Study*.

[33] Hockaday C., Crase S. J., Shelley M. C., Stockdale D. F. (2000). A prospective study of adolescent pregnancy. *Journal of Adolescence*.

[34] Mchunu G., Peltzer K., Tutshana B., Seutlwadi L. (2012). Adolescent pregnancy and associated factors in South African youth. *African Health Sciences*.

[35] Bandura A. (2012). On the functional properties of perceived self-efficacy revisited. *Journal of Management*.

[36] Tareq M. A., Khazaei H., Khazaei A. (2017). Emotional intelligence and charismatic leadership. *Innovation and Management*.

[37] Coyle K., Basen-Engquist K., Kirby D. (2016). Safer choices: reducing teen pregnancy, HIV, and STDs. Public health reports. *Department of “Population by States and Ethnic Group”. Ministry of Communications*.

[38] Ministry of Health Malaysia (2015). Perinatal care manual. *Ministry Of Health Malaysia*.

[39] Corcoran J. (2016). Teenage pregnancy and mental health. *Societies*.

[40] Mohd Arifin S. R., Ahmad A., Abdul Rahman R., Seng Loh H., Guan Ng C., Ng C. G. (2014). Postpartum depression in Malaysian women: the association with the timing of pregnancy and sense of personal control during childbirth. *International Journal of Academic Research*.

[41] Räisänen S., Sankilampi U., Gissler M. (2014). Smoking cessation in the first trimester reduces most obstetric risks, but not the risks of major congenital anomalies and admission to neonatal care: a population-based cohort study of 1 164 953 singleton pregnancies in Finland. *Journal of Epidemiology & Community Health*.

[42] Hernandez J., Abu Rabia H. M., Hazza M. (2017). Contributing factors to older teen mothers’ academic success as very young mothers. *International Journal of Higher Education*.

[43] Govender D. (2019). Teenage pregnancy and mental health. *Mental Health Matters*.

[44] Moni S. A., Nair M. K. C., Devi R. S. (2013). Pregnancy among unmarried adolescents and young adults. *Journal of Obstetrics & Gynaecology of India*.

[45] Oljira L., Berhane Y., Worku A. (2012). Pre-marital sexual debut and its associated factors among in-school adolescents in eastern Ethiopia. *BMC Public Health*.

[46] Higgins J. A., Lands M., Ufot M., McClelland S. I. (2022). Socioeconomics and erotic inequity: a theoretical overview and narrative review of associations between poverty, socioeconomic conditions, and sexual wellbeing. *The Journal of Sex Research*.

[47] Berry E. H., Shillington A. M., Peak T., Hohman M. M. (2000). Multi-ethnic comparison of risk and protective factors for adolescent pregnancy. *Child and Adolescent Social Work Journal*.

[48] Acharya D. R., Bhattarai R., Poobalan A., Teijlingen V. E., Chapman G. (2010). Factors associated with teenage pregnancy in South Asia: a systematic review. *Health Science Journal*.

[49] Allen E., Bonell C., Strange V. (2007). Does the UK government’s teenage pregnancy strategy deal with the correct risk factors? Findings from a secondary analysis of data from a randomised trial of sex education and their implications for policy. *Journal of Epidemiology & Community Health*.

[50] Hall W. J., Jones B. L., Witkemper K. D., Collins T. L., Rodgers G. K. (2019). State policy on school-based sex education: a content analysis focused on sexual behaviors, relationships, and identities. *American Journal of Health Behavior*.

[51] Mushwana L., Monareng L., Richter S., Muller H. (2015). Factors influencing the adolescent pregnancy rate in the greater Giyani municipality, Limpopo province – South Africa. *International Journal of Africa Nursing Sciences*.

[52] World Health Organization (2010). *Fact Sheet No 345, “Young People: Health Risks and Solutions*.

[53] Tabatabaie A. (2015). Childhood and adolescent sexuality, Islam, and problematics of sex education: a call for re-examination. *Sex Education*.

[54] Wong L. P. (2012a). An exploration of knowledge, attitudes and behaviours of young multiethnic Muslim-majority society in Malaysia in relation to reproductive and premarital sexual practices. *BMC Public Health*.

[55] Haldre K., Rahu K., Rahu M., Karro H. (2009). Individual and familial factors associated with teenage pregnancy: an interview study. *The European Journal of Public Health*.

[56] Nordin N., Wahab R. A., Yunus F. W. (2012). Psychological well-being of young unwed pregnant women: implications for extension education and programs. *Procedia-Social and Behavioral Sciences*.

[57] Farahani F. K. (2020). Adolescents and young people’s sexual and reproductive health in Iran: a conceptual review. *The Journal of Sex Research*.

[58] Hinkle W., Wiersma W. (2003). *Jurs. Applied Statistics for the Behavioral Sciences*.

[59] Khazaei H. (2019). The datasets of factors influencing adoption of electric cars in Malaysia: a structural equation modelling (SEM) analysis. *Data in Brief*.

